# Cytokine Production in the Serum and Spleen of Mice from Day 6 to 14 of Gestation: Cytokines/Placenta/ Spleen/Serum

**DOI:** 10.1155/1995/42412

**Published:** 1995

**Authors:** Irene Athanassakis, Bessy Iconomidou

**Affiliations:** Department of Biology University of Crete Heraklion Crete P.O. Box 1470 Greece

**Keywords:** Cytokines, pregnancy, maternal serum, spleen, placenta

## Abstract

Pregnancy, like most biologic phenomena, involves the action of cytokines. These proteins have a short half-life and are believed to exert their effect close to their site of production, where diagnostic tests cannot be easily performed. Here we show that the cytokine content in the maternal serum reflects cytokine production and secretion from maternal spleen cells, which also correlates with production from decidual cells. We show that GM-CSF, IL- 3, and IL-10 are present in the serum at specific time intervals during the first half of murine pregnancy, which correlates with their production from maternal spleen cells. Purified GM-CSF and IL-3 from spleen-cell-culture supernatants are biologically active molecules, able to stimulate placental-cell proliferation. Furthermore, TNF-0, which has been identified in many cases of fetal rejection as well as in labor, is shown to be naturally produced during the second half of pregnancy. Additionally, within the limits of the sensitivity of the technique we have used, the detection of IL-4 and the absence of detectable levels of IL- 2 in the maternal serum strongly comforts the hypothesis that pregnancy is a Th2-dependent phenomenon. The results presented in this paper show that the cytokine profile during pregnancy can be monitored by simple blood tests, which may be of relevance both in the followup of a physiological human pregnancy and to the diagnosis of recurrent abortions due to cytokine imbalance.


*Developmental Immunology*, 1996, Vol 4, pp. 247–255
Reprints available directly from the publisher
Photocopying permitted by license only

© 1996 OPA (Overseas Publishers Association)
Amsterdam B.V. Published in The Netherlands
by Harwood Academic Publishers GmbH
Printed in Singapore

### Cytokine Production in the Serum and Spleen of Mice from Day 6 to 14 of Gestation: Cytokines/Placenta/Spleen/Serum

IRENE ATHANASSAKIS* and BESSY ICONOMIDOU

*Department of Biology, University of Crete, P.O. Box 1470, 711 10 Heraklion, Crete, Greece*

Pregnancy, like most biologic phenomena, involves the action of cytokines. These proteins have a short half-life and are believed to exert their effect close to their site of production, where diagnostic tests cannot be easily performed. Here we show that the cytokine content in the maternal serum reflects cytokine production and secretion from maternal spleen cells, which also correlates with production from decidual cells. We show that GM-CSF, IL-3, and IL-10 are present in the serum at specific time intervals during the first half of murine pregnancy, which correlates with their production from maternal spleen cells. Purified GM-CSF and IL-3 from spleen-cell-culture supernatants are biologically active molecules, able to stimulate placental-cell proliferation. Furthermore, TNF-α, which has been identified in many cases of fetal rejection as well as in labor, is shown to be naturally produced during the second half of pregnancy. Additionally, within the limits of the sensitivity of the technique we have used, the detection of IL-4 and the absence of detectable levels of IL-2 in the maternal serum strongly comforts the hypothesis that pregnancy is a Th2-dependent phenomenon. The results presented in this paper show that the cytokine profile during pregnancy can be monitored by simple blood tests, which may be of relevance both in the followup of a physiological human pregnancy and to the diagnosis of recurrent abortions due to cytokine imbalance.

KEYWORDS: Cytokines, pregnancy, maternal serum, spleen, placenta.

### INTRODUCTION

During the gestational period, the maternal immune system is being stimulated by paternal alloantigens, which are introduced in the maternal organism through the semi-allogeneic fetus they carry. These alloantigens can be detected either in the maternal blood, where fetal cells have been found to circulate in mice and humans (Douglas et al., 1959; Schroder, 1975; Herzenberg et al., 1979; Bianchi et al., 1990; Bruch et al., 1991). In mice, they are expressed on the outer trophoblastic layer of the semi-allogeneic placental tissue, which comes in direct contact with the maternal circulation (McLaren, 1975; Raghupathy et al., 1981). Many studies so far indicate that at the cellular level, the maternal immune stimulation is manifested via two essential mechanisms. The first consists of an immunotrophic activity of the maternal immune system toward the developing feto-placental annexes (James, 1965; Wegmann, 1984; 1987), whereas the second involves an immunosuppressive activity of the maternal immune response toward paternal alloantigens (Slapsys and Clark, 1983, Chaouat et al., 1984; Sano et al., 1984). For a long time, the immunotrophic and immunosuppressive hypotheses were considered as mutually exclusive and scientists were trying to give evidence for either one. Regarding the immunotrophic hypothesis, there is evidence that maternal T cells and T-cell-derived lymphokines stimulate placental-cell growth and improve fetal survival (Wegmann, 1984; Athanassakis et al., 1987). On the other hand, the existence of anti-paternal suppressor factors and suppressor cells during pregnancy support the immunosuppressive hypothesis (Smith and Powell, 1977; Engleman, et al., 1978; Slapsys and Clark, 1983; Sano et al., 1984; Clark et al. 1990). How-

*Corresponding author.

ever, it is now believed that these two theories are not mutually exclusive and that both drive the maternal organism to cytokine production regulating important steps of successful pregnancy, which is hypothesized to be a Th2-dependent phenomenon (Wegmann et al., 1993).

Here we study six cytokines, referenced to be involved in the process of successful pregnancy. Interleukin-3 (IL-3) and granulocyte-macrophage colony-stimulating factor (GM-CSF) have been described to stimulate growth and function of trophoblasts (Athanassakis et al., 1987). Interleukin-10 (IL-10) shows a protective effect to the materno-fetal allograft, rescuing fetal rejection in abortion-prone mice (Chaouat et al., 1995). Tumor necrosis factor-α (TNF-α) has been detected in many cases of abortion models and its beneficial or destructive presence during pregnancy is questioned in many instances (Parand and Chedid, 1964; Gendron et al., 1989; Chaouat et al., 1990; Yelavarthi et al., 1991).

In order to distinguish between Th1 and Th2 cell populations, this study also included detection of interleukin-2(IL-2) and interleukin-4(IL-4) in the serum of pregnant mice, lymphokines that are specifically secreted by the two populations, respectively (Mossman et al., 1989).

To obtain as complete an estimate as possible of the bidirectional maternal immune reaction, it was decided to initially follow the positive regulatory signals transmitted by the maternal organism that are directed toward placental growth. Such positive reactions in the maternal spleen and decidual cap were indentified in previous studies by the production of pregnancy-specific growth factors in these tissues (Athanassakis-Vassiliadis 1992; Tsoukatos et al., 1994).

Because we aimed at designing a single method for the followup of pregnancy, we investigated, in a first step, whether the cytokine profile observed in the maternal serum from day 6 to 14 of gestation would reflect the cytokine production in the maternal spleen. We show that this is indeed the case, and confirm that the production of trophoblast-stimulating lymphokines is a systemic phenomenon. Therefore, it could be followed during pregnancy by simple blood tests. Such an observation could be very useful in clinical situations, if confirmed in humans.

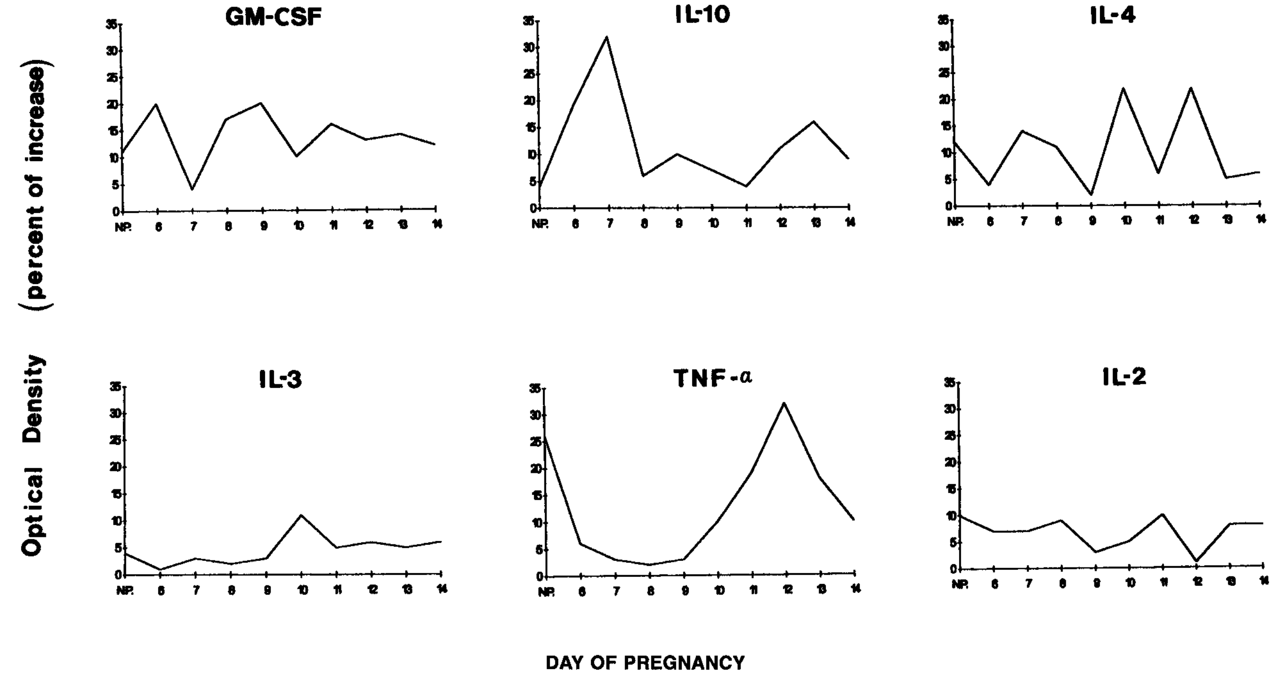

FIGURE 1. Analysis of GM-CSF, IL-3, IL-10, TNF-α, IL-2, and IL-4 in the serum of virgin or pregnant mice from day 6 to 14 of gestation by ELISA. The results are expressed as the percentage of optical density above background levels. The values corresponding to virgin mice are shown at the beginning of the axes (NP). The results represent the mean of three mice tested in duplicates. Each experiment was repeated three times and comparable results were obtained. In all cases, standard deviations do not exceed 3%.

### RESULTS

### Detection of IL-2, IL-3, IL-4, IL-10, GM-CSF, and TNF-α in the Serum of Pregnant Mice

Cytokine balance is now believed to play an important role in the maintenance of pregnancy. In order to define the physiological levels of IL-2, IL-3, IL-4, IL-10, GM-CSF, and TNF-α, blood was collected from mice from day 6 to 14 of gestation and the serum was analyzed for the content of various cytokines by ELISA. For each day of pregnancy, 3 mice were sacrificed and their sera were run 3 times in duplicates. The results, which represent the mean of all the experiments, are shown in Fig. 1.

GM-CSF is showing a steady production from day 6 to 14 of gestation, where day-7 sera contain the lowest amount of the factor and day 9 the highest; IL-3 is detected in the serum after the tenth day of pregnancy. IL-10 shows a peak of production on day 7 of pregnancy, which declines at lower levels until the fourteenth day; TNF-α increases after the second half of pregnancy reaching a peak of production on the twelfth day; IL-2 is found at low levels throughout the days of pregnancy tested, which do not differ from the IL-2 production observed in nonpregnant mice (in the same experiments, IL-2 could be detected in ConA-stimulated spleen-cell-culture supernatants — data not shown); IL-4 is generally present in the serum of pregnant mice at higher amounts than nonpregnant mice and shows two peaks of production on days 10 and 12 of pregnancy.

### Detection of IL-3, IL-10, and GM-CSF in the Spleen of Pregnant Mice

Previous studies have shown that specific growth-factor production during pregnancy takes place not only at the proximity of the feto-placental unit-decidual cap (Athanassakis-Vassiliadis, 1993), but in the maternal spleen as well (Tsoukatos et al., 1994). Therefore, similar analysis was performed on the spleen cells of pregnant mice from day 6 to 14 of gestation, where the percentage of cytokine-secreting cells was estimated by intracellular immunofluorescence. The results, which represent the same number of mice and experiments mentioned in the previous section, are shown in Fig. 2.

GM-CSF-secreting cells are showing a constant pattern from day 6 to 14 of gestation, which correlates with the appearance of this factor in the serum, with an exception on day 7 of pregnancy, when the cellular factor peaks and the equivalent seric content markedly decreases; IL-3-secreting cells are detected in the spleen only on days 6 and 7 of gestation, which indicates a cessation of its production thereafter; IL-10-secreting cells show a similar peak of stimulation on day 7 of pregnancy, a pattern that correlates with the appearance of this factor in the serum.

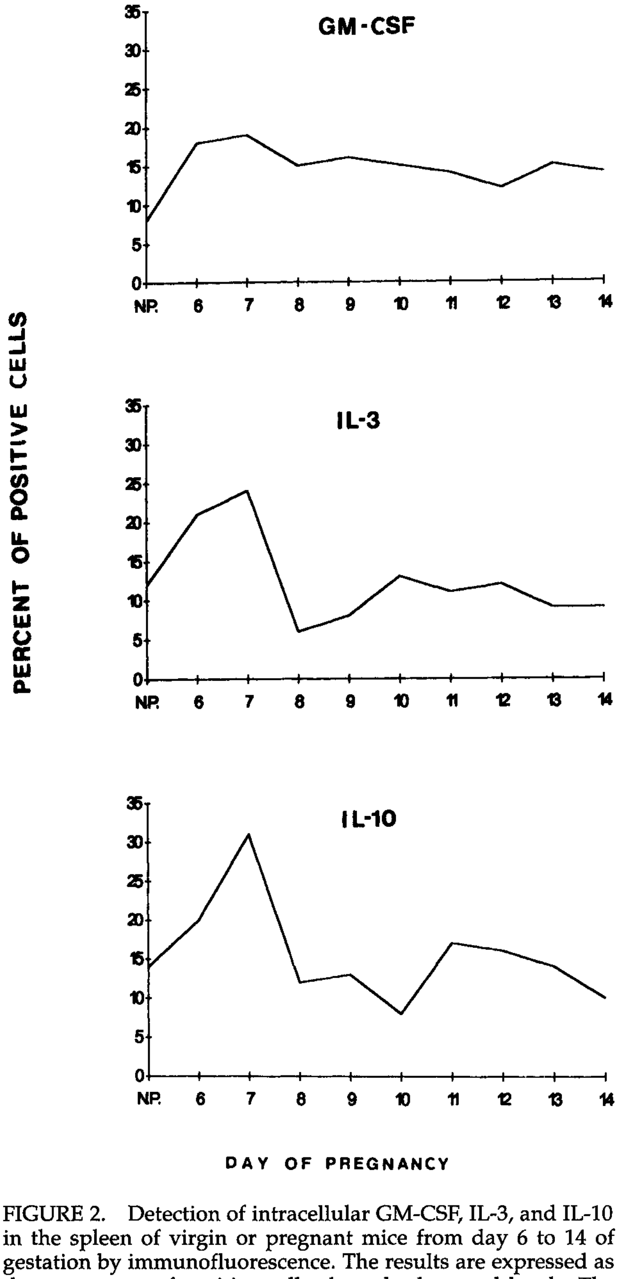

FIGURE 2. Detection of intracellular GM-CSF, IL-3, and IL-10 in the spleen of virgin or pregnant mice from day 6 to 14 of gestation by immunofluorescence. The results are expressed as the percentage of positive cells above background levels. The values corresponding to virgin mice are shown at the beginning of the axes (NP). The results represent the mean of three mice tested in duplicates. In all cases, standard deviations do not exceed 2%.

### Spleen-Cell-Culture Supernatants from Gestating Mice Contain GM-CSF and IL-3

Among the cytokines tested so far, special care is given to GM-CSF and IL-3, which are known to fortify the placental barrier by stimulating trophoblast growth and phagocytosis (Athanassakis et al., 1987). The results presented in the previous section show that indeed GM-CSF- and IL-3-producing cells are present in the spleen. In order to test whether these lymphokines also can be found in secreted form, we collected 24-hr-culture supernatants from mice at different days of pregnancy, purified them through affinity columns, and tested the presence of GM-CSF and IL-3 in the resulting column fractions by ELISA. Thus, pregnant mice from day 9 to 13 of gestation (three mice for each day of pregnancy) were sacrificed, the spleens were removed, put in cell suspension, and cultured for 24 hr in a serum-free culture medium. The culture supernatants were individually loaded on the top of a Heparin Sepharose CL-6B affinity column and eluted with a gradient of NaCl (0.1 to 1.1 M). The resulting fractions were dialyzed against PBS, their protein content determined, and the same amount of protein from each fraction was assayed for the presence of GM-CSF and IL-3. As shown in Fig. 3, GM-CSF is detected in fractions eluted with 200–700 mM NaCl and maximal values are obtained on days 9 and 11. In nonpregnant mice, no GM-CSF could be detected in any of the fractions of the spleen-cell supernatants, indicating that the increased production of GM-CSF is related to the gestating cycle of the mother.

IL-3 was eluted with 200–600 mM NaCl. This lymphokine is detected on days 9, 10 and 11 of pregnancy (Fig. 3). Although virgin females show some production of this factor (10% increase over background), during pregnancy, IL-3 doubles its production.

### Spleen-Cell-Culture Supernatants from Gestating Mice Contain Proteins Stimulating Placental Cell Growth

In order to test whether the growth factors contained in the various affinity column fractions of spleen-cell supernatants may influence placental-cell growth, we applied purified supernatants to placental cells and assayed 4 days later for cell proliferation by $^{3}$HTdR uptake. The results show that indeed biologic activity can be detected in various column fractions (Fig. 4). The general pattern of biologic activity can be divided in four peaks according to the NaCl concentration used for elution: (1) 200–400, (2) 400–600, (3) 600–800, and (4) 800–1000 mM NaCl. Peak number 1 was active only on the tenth day of pregnancy, when it showed a twofold increase in placental-cell proliferation over background. The second peak gave an increasing activity from day 9 of pregnancy, reaching highest levels on the eleventh day and showing only background activity on the thirteenth day of syngeneic pregnancy. Peak 3 gave the highest levels of biologic activity on the twelfth day of pregnancy (sevenfold). With the fourth peak, an increasing biologic activity was observed from day 9 of pregnancy, reaching maximal levels by day 12. Although the peaks of activity seen on days 9, 10, and 11 may correspond to the

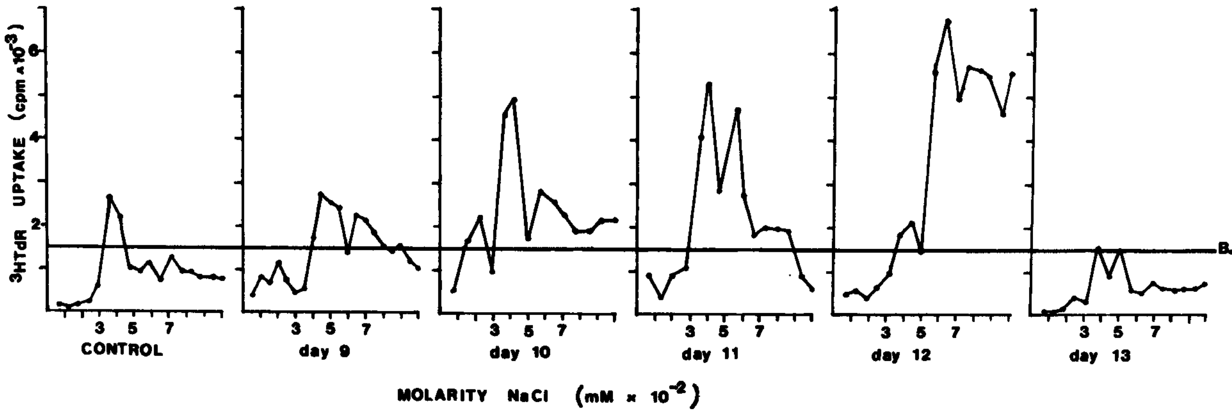

FIGURE 3. Analysis of affinity column fractionated spleen-cell-culture supernatants in pregnant mice from day 9 to 13 of gestation. Biologic activity was determined as the ability of the resulting factors to stimulate placental cell proliferation (see Materials and Methods). The base line (B.L.) represents the background level of placental-cell proliferation.

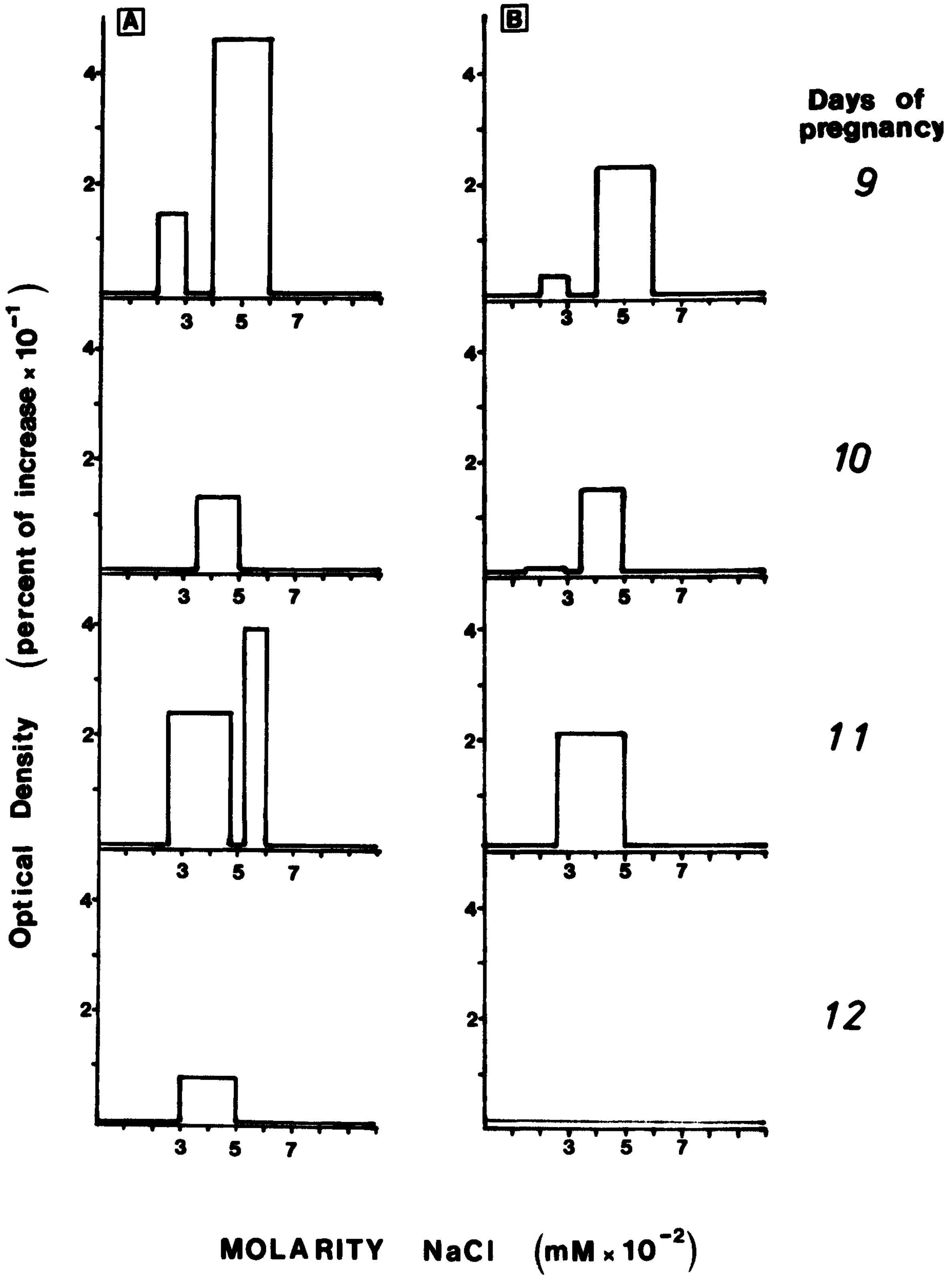

FIGURE 4. Analysis of GM-CSF (A) and IL-3 (B) content in the affinity column fractionated spleen-cell-culture supernatants in pregnant mice. This analysis was performed using the ELISA technique (see Materials and Methods) and the results are expressed as the percentage of optical density above background levels. GM-CSF could not be detected in fractionated spleen-cell-culture supernatnats from nonpregnant controls, whereas IL-3 content in the fractionated spleen-cell-culture supernatants from nonpregnant controls showed a 10% activity over background.

already defined GM-CSF and IL-3 growth factors, the results show that additional placental growth factors are secreted on day 12 of pregnancy.

### DISCUSSION

During the physiological process of pregnancy, the fetal allograft develops in a restricted site of the maternal body, the uterus, which is somehow shed from the rest of the maternal organism. Important regulatory maternal/fetal interactions take place at the uterine site, where fetal annexes come in direct contact with the maternal circulation. From the moment of fertilization, a plethora of growth factors produced in the uterus harmonically exert their action to ensure the development of the fetal allograft (Hill, 1991; Lea et al., 1992; Lin et al., 1993; Delassus et al., 1994). However, the uterus is not the only site affected by fetal growth. Changes also occur in the rest of the maternal organism that should play an equally important role in fetal growth and survival. Previous studies have shown that spleen cells on the eleventh day of pregnancy produce placenta-specific growth factors, which are not seen in virgin females (Tsoukatos et al., 1994). In order to examine whether such cytokine changes can be detected in the maternal serum and whether these reflect the situation observed at specific sites of the maternal organism, in the present study; we compared the cytokine content in the maternal serum from day 6 to 14 of gestation with the growth factors produced and secreted by spleen cells within the same time intervals. The results demonstrate that the cytokine profile in the serum correlates with that of maternal spleen and furthermore with the local production of growth factors by decidual cells (Athanassakis-Vassiliadis, 1993).

The profile of GM-CSF, IL-3, IL-10, TNF-α, IL-2, and IL-4 in the maternal serum was determined by ELISA. GM-CSF and IL-3 are known to directly affect fetal development by stimulating trophoblast proliferation, whereas IL-10 has been shown to rescue fetal abortion in mice (Chaouat et al., 1995). The results show that the production of these lymphokines is not simultaneous, because IL-10 peaks on day 7 of pregnancy, GM-CSF on day 9, and IL-3 on day 10. Consequently, it can be postulated that these factors serve different stages of fetal development. The presence of TNF-α has been correlated with fetal abortion in many systems (Parand and Chedid, 1964; Gendron et al., 1989; Chaouat et al., 1990). Here we show that TNF-α is present in the maternal serum after the second half of pregnancy and its production peaks on day 12. This observation places TNF-α among the cystostatic factors induced later in pregnancy, which are necessary to restore equilibrium via-à-vis the stimulatory factors produced at the beginning of the gestational period.

The presence of these factors in the maternal serum places once more T cells in the first line of interest (Heyborn et al., 1992), because under physiological conditions, these consist of the primary source of the earlier proteins. In an attempt to distinguish between Th1 and Th2 subpopulations, we determined the IL-2 and IL-4 content of the maternal serum, lymphokines known to be specifically produced by the two cell types, respectively. Although the amounts of IL-2 do not differ from those of virgin mice, the production of IL-4 was found to increase during pregnancy, showing two peaks on days 10 and 12, results arguing for a Th2-dependent pregnancy process, as it has been postulated by Wegmann et al. (1993).

In order to define whether spleen is one of the sites of production of such growth factors, we stained maternal spleen cells with specific monoclonal antibodies and showed that indeed IL-10-secreting cells are present in the maternal spleen, following a profile similar to that of maternal serum. Intracellular GM-CSF, which with an exception on day 7 of gestation follows a similar pattern to the seric factor, is also being secreted by spleen cells because it can be detected in the 24-hr culture supernatants of spleen cells at the equivalent days of pregnancy. The highest concentration of IL-3-secreting cells is detected in the spleen of days 6 and 7 of pregnancy, which, however, does not correspond to the highest concentration of the factor detected in the serum. This form of IL-3 is either nonsecreted or it is at the beginning of its biosynthesis, because IL-3 can be detected in the 24-hr culture supernatants from day 9 of pregnancy. Furthermore, GM-CSF and IL-3 secreted by spleen cells are biologically active because they can stimulate placental cells to proliferate. The differences observed between seric and intracellular growth factors can be due to the presence of soluble receptors, to different sites of action as well as to the difference in factor half-life, points that need to be investigated in the future. The factors studied here are naturally not the only ones involved in the gestational process. Indeed, when fractions of the 24-hr-spleen-cell culture supernatants, other than IL-3 and GM-CSF, were applied to placental cells, these were able to stimulate growth.

These results give new insight in the regulatory mechanisms taking place during pregnancy. Each growth factor follows a specific pattern of production, likely to serve specific needs of the materno/fetal organism during gestation. One important observation is that growth-factor production is a systemic phenomenon during pregnancy and that its presence in the maternal serum correlates with its production from spleen and possibly other sites close to the feto-placental unit. A similar method as the one used for the murine growth-factor profiles analyzed here may be employed in humans, to allow to study the physiological levels of cytokines throughout the gestational period. Several abortive causes have been correlated to specific growth factor over or under production. Therefore, with simple blood tests, we may follow not only the progress of fetal development, but we can also prognose cases of recurrent abortion.

### MATERIALS AND METHODS

### Mice

BALB/cJ(H-$2^d$) 6–8 weeks old were maintained in the animal facility at the University of Crete (Crete, Greece). Each female was checked for oestrus, caged overnight with a BALB/cJ male, and checked for the presence of a vaginal plug on the following morning. The day on which the plug was observed was considered to be day 0 of pregnancy.

### Antibodies

Rat anti-mouse IgG2a monoclonal antibodies to the growth factors GM-CSF (sensitivity 5 μg/ml), IL-3 (sensitivity 3 μg/ml), IL-10 (sensitivity 0.14 U/ml), TNF-α (sensitivity 25 ρg/ml), IL-2 and IL-4 (sensitivity 5 μg/ml) were purchased from Endogen Inc. (Cambridge, MA), and were used at the concentration of 1 μg/ml.

### Spleen-Cell Cultures

Spleens were obtained either from pregnant or control nonpregnant BALB/cJ females from day 9 to 13 of gestation. Cell suspensions were washed in Hank's solution (Gibco, Grand Island, NY) three times and cultured in RPMI 1640 medium (Gibco) without serum for 24 hr at a concentration of $1 \times 10^6$ cells/ml. Culture supernatants (10 ml) were collected after centrifugation of the cells at 1200 rpm for 10 min. Three mice were tested in each day of pregnancy in the allogeneic and syngeneic situation.

### Partial Fractionation of Spleen-Cell Supernatants

This fractionation was performed as described by Tsoukatos et al. (1994) with slight modifications. Briefly, three samples of 10 ml spleen cell supernatant, obtained from three different mice were pooled, dialyzed against PBS, and passed through a Heparin Sepharose CL 6B affinity column. The column was equilibrated with buffer A (20 mM Tris, pH 7.4, 100 mM NaCl) following a flow rate of 5 ml/hr. The column was then washed with buffer A, loaded with the sample and a gradient (50 ml) of 0 to 1 M NaCl was applied. Fractions of 5 ml were collected, dialyzed against PBS, and kept at 4°C until assayed on placental cell proliferation.

### Placental Cell Cultures

Placental cell cultures were performed as described by Athanassakis et al. (1987) with slight modifications. Briefly, placentas were isolated from females on day 11 to 14 of gestation and after removing the maternally derived decidual cap layer from all placentas, the tissues were cut in small pieces, and single-cell suspensions were prepared by passing the preparation through a syringe with a $18\frac{1}{2}$ G needle, into Hank's solution (Athanassakis et al. 1987; 1989). As previously reported, this is a very mild technique for isolating trophoblasts from the murine placenta, yielding 95 to 100% viable cells. The cell types isolated by the method used include the adherent spongiotrophoblasts (19±2%), labyrinthine trophoblasts (69±8%), macrophages (3±1%), and red cells. $^3$H-Thymidine ($^3$HTdR) incorporation assays followed by autoradiography demonstrate that only the adherent placental cells proliferate in a cell culture (Athanassakis et al., 1993), which are also found to be cytokeratin-positive and vimentin-negative (trophoblasts; Athanassakis, 1987). For each experiment, placental cells from 3 to 4 mice (pool of 20 to 24 different placentae) were isolated as described before, washed three times, and cultured at the concentration of $1 \times 10^7$ cells/ml in RPMI 1640 medium supplemented with 20% fetal calf serum (FCS, Seralab, Sussex), in 96-well plates (Linbro, McLean,

VA) with or without the presence of the fractionated spleen-cell supernatants at a final volume of 200 μl/well in triplicates. Protein fractions from the Heparin column were used at 1:2 dilution. Cultures were assayed for $^{3}$H-Thymidine $^{3}$HTdR, NEN, Du Pont, Paris) incorporation on day 4. This time period was found to be the most appropriate to placental cell cultures, where all cells have gone through two cell cycles and maintain their full viability (Athanassakis, 1993). One μCi of $^{3}$HTdR was added per well 18 hr prior to harvest. The cells were collected with vacaum aspiration on cellulose filters. By using an automatic titertec cell harvester (Flow Labs), free $^{3}$HTdR was washed away with repeating flashes of $ddH_2O$. The filters were air dried, placed in scintillation fluid (toluene supplemented with 1.38 g/l omnifluor, NEN), and counted in a LKB 1218 Rackbeta counter. These experiments were repeated three times for each set of column fractions, for each day of pregnancy tested, and gave comparable results.

### Indirect ELISA

Mouse serum, collected from virgin or pregnant mice from day 6 to 14 of gestation, was used at the dilution of 1/1000 in carbonate buffer pH 9.6. Protein concentration (mg/ml) in the different column fractions was estimated by measuring the optical density at 280 and 260 nm using a spectrophotometer (Hitachi K-1100) and applying the formula: (1.45 × Absorption 280) – (0.74 × Absorption 260). Each fraction was coated at the concentration of 5 μg/ml in carbonate/bicarbonate buffer, pH 9.6. All serum samples or fractionated spleen-cell-culture supernatants were coated in the same 96-well ELISA plate (Nunc, Kampstrup, Denmark), incubated overnight at 4°C, and washed four times in 5% Tween-20. The remaining sites were blocked by a 2% PBS-BSA solution after incubation for 2 hr at room temperature. After washing four times as described before, 100 μl of the different antibodies, diluted in 0.1% PBS-BSA, were added and incubated for 1 hr at room temperature. After washing, 100 μl of goat anti-mouse IgG coupled to horseradish peroxidase (1:1000 dilution, Sigma) was added and incubated for 1 hr at room temperature, in the dark. The plate was then washed and 100 μl/well of tetramethyl benzidine-$H_2O_2$ (Sigma) were added and incubated for 20 min. The enzymatic reaction was stopped by adding 50 μl $H_2SO_4$ (4N). Optical density (OD) was measured at 450 nm using a Titertec ELISA photometer (Multiskan Plus). The ELISA test was performed at least three times for each set of affinity-purified column fractions. The results were expressed as the percentage of OD increase over background (OD of antibody alone).

### Indirect Immunofluorescence

Immunofluorescent staining of cytoplasmic growth-factor production was performed as described by Sander et al. (1991). Maternal spleen cells from day 6 to 14 pregnancy were put in a single-cell suspension, washed several times, and fixed with ice-cold paraformaldehyde (4% in PBS) for 5 min. After washing, the cells were incubated for 30 min at room temperature with specific antibodies diluted in a HBSS-Saponin solution (HBSS; Gibco, 0.01 M Hepes; Gibco, 0.1% Saponin, Sigma). After washing in PBS-Saponin, the cells were incubated with rabbit anti-rat IgG antibody FITC-conjugated for 30 min at room temperature. The cells were washed, fixed with 25% glycerol, mounted on slides, and examined for cytoplasmic staining using a Leitz fluorescent microscope.

### ACKNOWLEDGMENTS

We thank D. Tsoukatos and G. Skarpelis for technical assistance. This work was supported by University of Crete funds.

*(Received July 20, 1995)*

*(Accepted September 11, 1995)*

### REFERENCES

Athanassakis I., Bleackley, R.C., Paetkau V., Guilbert L., Barr P.J., and Wegmann, T.G. (1987). The immunostimulatory role of T cells and T cell-derived lymphokines on murine fetally-derived placental cells. J. Immunol. **138**: 37.

Athanassakis I., Papamatheakis J., and Vassiliadis S. (1993). Specific CSF-1 binding on murine placental trophoblasts and macrophages serves as a link to placental growth. J. Recept. Res. **13**: 739.

Athanassakis-Vassiliadis I., Thanos D., and Papamatheakis J. (1989). Induction of class II major histocompatibility antigens in murine placenta by 5-azacytidine and interferon-γ involves different cell populations. Eur. J. Immunol. **19**: 2341.

Athanassakis-Vassiliadis I., Vassiliadis S., and Papamatheakis J. (1992). Common mechanisms govern the expression of p21ras and class II MHC antigens in the murine placenta. J. Reprod. Immunol. **21**: 149.

Athanassakis-Vassiliadis I. (1993). Lymphokine production by decidual cells in allogeneic and syngeneic murine pregnancy. Cytokine 4: 354.

Bianchi D.W, Flint A.F., Pizzimenti M.F., et al. (1990). Isolation of fetal DNA from nucleated erythrocytes in maternal blood. Proc. Natl Acad. Sci. USA. **87**: 3279.

Bruch J.F., Metezeau, P., Garcia-Fonknechten N., et al. (1991). Trophoblast-like cells sorted from peripheral blood using flow cytometry: A multiparametric study involving transmission electron microscopy and foetal DNA amplification. Prenat. Diag. **11**: 787.

Chaouat G., Kolb J.P., and Riviere M. (1984). Role of the placental interface and the trophoblast/maternal tissue interactions in the survival of the murine fetal allograft. Ann. Immunol. (Paris) **135D**: 302.

Chaouat G., Meliani A.A., Martal J., Raghupathy R., Elliot J., Mosmann T., and Wegmann T.G. (1995). IL-10 prevents naturally occurring fetal loss in the CBA × DBA/2 mating combination, and local defect in IL-10 production in this abortion-prone combination is corrected by in vivo injection of IFN-τ. J. Immunol. **154**: 4261.

Chaouat G., Menu E., Clark D.A., Dy M., Minkawski M., and Wegmann T.G. (1990). Control of fetal survival in CBA×DBA/2 mice by lymphokine therapy, J. Reprod. Fertil. **89**: 447.

Clark D.A., Flanders K.C., Banwatt D., Millar-Book W., Manuel J., Stedronska-Clark J., and Rowley B. (1990). Murine pregnancy decidua produces a unique immunosuppressive molecule related to transforming growth factor β-2. J. Immunol. **144**: 3008.

Delassus S., Coutinho, G.C., Saucier C., Darche S., and Kourilsky P. (1994). Differential cytokine expression in maternal blood and placenta during murine gestation. J. Immunol. **152**: 2411.

Douglas G.W., Thomas L., and Carr M. (1959). Trophoblasts in the circulating blood during pregnancy. Amer. J. Obstet. Cynecol. **78**: 960.

Engelman E.G., McMichael A.J., and McDevitt H.O. (1978). Suppression of the mixed lymphocyte reaction in man by a soluble T cell factor: Specificity of the factor for both the responder and the stimulator. J. Exp. Med. **147**: 1037.

Gendron R.L., Nestel F.P., Lapp W.S., and Baines M.G. (1989). Lipopolysaccharide induced fetal resorption involves embryo associated production of Tumor Necrosis Factor. *In*: Proceedings of the 7th International Congress Immunology, Berlin. (Abstract 118/017.)

Herzenberg L.A., Bianchi, D.W., Schroder J., et al. (1979). Fetal cells in the blood of pregnant women: Detection and enrichment by fluorescence-activated cell sorting. Proc. Natl Acad. Sci. USA. **76**: 1453.

Heyborn K.D., Cranfill R.L., Carding S.R., Born W.K., and O'Brien R.L. (1992). Characterisation of γδ T lymphocytes at the maternal-fetal interface. J. Immunol. **149**: 2872.

Hill J.A. (1991). Implications of cytokines in male and female sterility. *In*: Cellular and molecular biology of the materno-fetal relationship, Chaouat G., and Mowbray J., Eds. (Paris: Colloque INSERM/John Libbey Eurotext Ltd., pp. 269–275.)

James D.A. (1965). Effects of antigenic dissimilarity between mother and fetus on placental size in mice. Nature **205**: 613.

Lea R.G., Flanders K.C., Harley C.B., Manuel J., Banwatt D., and Clark D.A. (1992). Release of a transforming growth factor (TGF)-β2-related suppressor factor from postimplantation murine decidual tissue can be correlated with the detection of a subpopulation of cells containing RNA for TGF-β2. J. Immunol. **148**: 778.

Lin H., Mossman T.R., Guilbert L., Tuntipopipat S., and Wegmann T.G. (1993). Synthesis of T Helper-2 cytokines at the maternal fetal interface. J. Immunol. **151**: 4562.

McLaren A. (1975). Antigenic disparity: Does it affect placental size, implantation or population genetics. *In*: Immuobiology of trophoblast, Edwards R.G., Howe C.W.S., and Johnson M.H., eds (Cambridge: Cambridge University Press), p. 225.

Mosmann T.R., and Coffman R.L. (1989). TH1 and TH2 cells: Different patterns of lymphokine secretion lead to different functional properties. Annu Rev. Immunol. **7**: 145.

Parand M., and Chedid L. (1964). Protective effects of chlorpromazine against endotoxin induced abortion. Proc. Soc. Exp. Biol. Med. **116**: 906.

Raghupathy R., Singh B., Barrington-Leigh J., and Wegmann T.G. (1981). The ontogeny and turnover kinetics of paternal H-2K antigenic determinants of the allogeneic murine placenta. J. Immunol. **127**: 2074.

Sander B.J., Anderson J., and Anderson U. (1991). Assessment of cytokines by immunofluorescence and the paraformaldehyde-saponin procedure. Immunol. Rev. **119**: 65.

Sano M., Miake S., Yashikai Y., and Nomoto K. (1984). Existence of suppressor cells in the spleen of alogeneic and syngeneic primiparous pregnant mice. J. Reprod. Immunol. **6**: 239.

Schroder J. (1975). Transplacental passage of blood cells. J. Med. Genet. **12**: 230.

Slapsys R., and Clark D.A. (1983). Active suppression of host-versus-graft reaction in pregnant mice. V. Kinetics, specificity and in vivo activity of non-T suppressor cells localised to the genital tract of mice during first pregnancy. Am. J. Reprod. Immunol. **3**: 65.

Smith R.N., and Powell A.E. (1977). The transfer of pregnancy induced hyporesponsiveness to male skin grafts with thymus dependent spleen cells. J. Exp. Med. **156**: 899.

Tsoukatos D., Skarpelis G., and Athanassakis I. (1994). Placenta-specific growth factor production by splenic cells during pregnancy. Placental **15**: 467.

Wegmann T.G. (1984). Fetal protection against abortion. Ann. Immunol. (Paris) **135D**: 309.

Wegmann T.G. (1987). Placental immunotrophism: Maternal T cells enhance placental growth and function. Amer. J. Reprod. Immunol. Microbiol. **15**: 67.

Wegmann T.G., Guilbert L.J., and Mossmann T.R. (1993). Bidirectional cytokine interactions in the maternal-fetal relationship: Successful pregnancy is a Th2 phenomenon. Immunol. Today **14**: 353.

Yelavarthi K.K., Chenm H.-L., Yang Y., Cowley B.D., Fishback J.L., and Hunt J.S. (1991). Tumor necrosis factor-α mRNA and protein in rat uterine and placental cells. J. Immunol. **146**: 3840.

